# The Aryl Hydrocarbon Receptor Relays Metabolic Signals to Promote Cellular Regeneration

**DOI:** 10.1155/2016/4389802

**Published:** 2016-08-03

**Authors:** Fanny L. Casado

**Affiliations:** Laboratorio de Investigación en Ciencias Ómicas y Biotecnología Aplicada, Pontificia Universidad Católica del Perú, Avenida Universitaria 1801, San Miguel, Lima 12, Peru

## Abstract

While sensing the cell environment, the aryl hydrocarbon receptor (AHR) interacts with different pathways involved in cellular homeostasis. This review summarizes evidence suggesting that cellular regeneration in the context of aging and diseases can be modulated by AHR signaling on stem cells. New insights connect orphaned observations into AHR interactions with critical signaling pathways such as WNT to propose a role of this ligand-activated transcription factor in the modulation of cellular regeneration by altering pathways that nurture cellular expansion such as changes in the metabolic efficiency rather than by directly altering cell cycling, proliferation, or cell death. Targeting the AHR to promote regeneration might prove to be a useful strategy to avoid unbalanced disruptions of homeostasis that may promote disease and also provide biological rationale for potential regenerative medicine approaches.

## 1. Understanding Self-Renewal Is Critical to Study Tissue Regeneration

Cellular regeneration involves the sum of events that renew cells, restore their function, and sustain their growth. The signals that trigger cellular regeneration can be physiological, such as cellular turnover or aging, or pathological, such as trauma or disease. Cellular regeneration is not a new field of scientific inquiry, but during the past decades it has been enriched with new insights and tools brought by stem cell research leading to the development of regenerative medicine. Regenerative medicine aims to replace, engineer, or regenerate human cells, tissues, or organs with the final goal of restoring or establishing normal function [1]. A vital connection between stem cell function and cellular regeneration is the concept of self-renewal in adult and postnatal stem cells. Self-renewal allows maintenance of the hierarchical structures of tissues when the potential of the parental cell to proliferate and differentiate is retained in one or both progenies. Thus, self-renewal is an asymmetrical type of cell division where all or some of the progeny maintains the proliferation and differentiation status of the parental cell [2].

Nowadays, genomic and proteomic approaches can be used to evaluate whether self-renewal pathways are involved in the development of disease, aging, or exposure to chemicals. However, the most robust approach available to test self-renewal functionally is the limiting dilution assay [3]. These assays have been exquisitely established in mouse models but their application in tissues other than blood and mammary gland may suffer from some limitations because the quantitation requires working with a large range of number of donor cells that may not be realistic. This assay is used to calculate the number of cells that have the ability to regenerate recipients of transplantations as a proportion of donor cells. At the beginning of the study, at least three different numbers of donor cells are tested. At the end of the experiment, the number of positively or negatively regenerated recipients is scored. The results are analyzed assuming that (1) only self-renewing cells regenerate the recipients, (2) one rare and randomly dispersed donor cell with self-renewing ability is enough to regenerate the recipient [4], and (3) the probability of regeneration follows a single-hit model of the Poisson distribution [5]. In addition, there are different in vitro serial dilution assays but since they may not recapitulate the entire tissue hierarchy, caution needs to be used when drawing conclusions about self-renewal from them. Thus, when weighting the evidence about the involvement of certain pathways in self-renewal and maintenance of tissue homeostasis, we need to take into consideration the resolution and power of the techniques used to generate the data.

For an asymmetrical cell division to occur, some cues are governed intrinsically by organismal programs [6]. Alternatively, there can be extrinsic cues contributing to self-renewal that have been abundantly demonstrated and account for a significant flexibility in the regulation of self-renewal [7]. For instance, in the absence of cell contact, conditioned media from stromal cells in combination with the steel factor and interleukin 11 can support self-renewal of hematopoietic stem cells (HSC) [8]. This flexibility opens up opportunities in regenerative medicine to promote tissue regeneration by tapping into the signaling responsible for extrinsic self-renewal control.

## 2. The Role of AHR in Self-Renewal of Stem Cells Is Cell Context Specific and It Is Mediated by Well-Established Self-Renewal Signaling

Evidence accumulated for the past 30 years supports that the aryl hydrocarbon receptor (AHR) is not only a xenobiotic sensor but that it also promotes changes in homeostasis of tissues whose functions rely on the maintenance of heterogeneously differentiated populations derived from self-renewing stem cells.

Historically, the AHR was discovered as the mediator of the toxic responses of halogenated aromatic hydrocarbons and polycyclic aromatic hydrocarbons, including its most potent known exogenous ligand 2,3,7,8-tetrachlorodibenzo-p-dioxin (TCDD) [9]. Canonical ligand binding to AHR occurs in the cytosol leading to a sequence of conformational changes that ultimately transform the AHR into its high-affinity DNA binding form in the nucleus to function as a transcription factor together with its heterodimeric partner HIF1*β* [10]. A TCDD-binding fingerprint of the conserved residues threonine 283, histidine 285, phenylalanine 289, tyrosine 316, isoleucine 319, phenylalanine 345, and alanine 375 within the ligand binding domain of the AHR forms a cavity conserved in mammalian AHRs necessary for optimal high-affinity TCDD binding [11]. Canonical AHR signaling induces drug-metabolizing enzymes (CYP1A1, CYP1A2, and CYP1B1), the glutathione-S-transferase, NQO1, and ALDH3 [12]. Functional studies in animal models of acute and developmental exposure to the AHR agonist TCDD [13, 14] show a direct detrimental effect in the self-renewing capacity of murine HSC. Changes in hematopoietic progenitor cell numbers have been observed in vitro when treatment with the AHR agonist benzo(a)pyrene led to decreases in the numbers of phenotypically defined human hematopoietic progenitors (CD34^+^) [15] and increases in CD34^+^ when exposed to the AHR antagonists StemRegenin-1 (SR1) [16] and GNF351 [17]. In addition, AHR antagonism with SR1 has been shown to be an efficient strategy to increase the numbers of CD34^+^ by increasing self-renewal in human cells even in the context of pathological conditions such as leukemia and genetic instability [18]. Furthermore, the role of AHR signaling in self-renewal has been shown due to AHR's interaction with the RNA-binding protein MSI2 [19]. The effect of xenobiotic AHR antagonists to increase stem cells populations was an inflection point in our understanding about AHR and self-renewal because, after these findings, research on AHR went beyond the toxicology field to revitalize efforts in regenerative medicine dealing with novel ways to increase the quantity while maintaining the quality of stem cells for cellular therapy applications [20].

**Figure 1:**
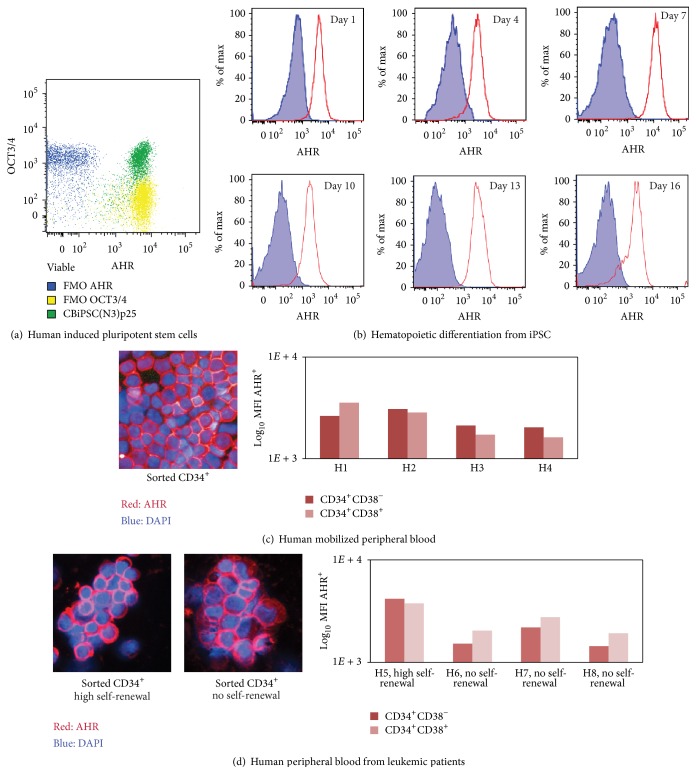
Ubiquitous AHR expression in iPSC and HSC regardless of potency and self-renewal activity. (a-b) AHR is expressed in (a) iPSC (OCT3/4+) and (b) cells from iPSC-derived embryoid bodies differentiating into hematopoietic lineages. Blue dots/areas correspond to fluorescence minus one (FMO) gating controls. (c-d) AHR is expressed in mobilized peripheral blood which is known to be enriched for (c) HSC and (d) leukemic cells with abnormal self-renewing activity.

While there is a general consensus that AHR expression is ubiquitous in prenatal and adult tissues, it was not clear whether AHR levels in vitro and throughout loss of pluripotency may change. To evaluate AHR expression on an in vitro developmental model, cord blood cells were reprogrammed into induced pluripotent stem cells (iPSC) and stained for the marker of pluripotency OCT3/4 and AHR. Next, embryoid bodies were formed from iPSC and differentiated into hematopoietic lineages as previously described [21]. Flow cytometry was used to analyze AHR expression using the commercially available AHR antibody (clone RPT9) conjugated in house to allophycocyanin. As expected, AHR is expressed in undifferentiated iPSC (Figure 1(a)), and the expression is maintained even after embryoid body-mediated hematopoietic differentiation in vitro (Figure 1(b)). Furthermore, AHR is expressed in human hematopoietic stem/progenitor cells and localized mostly within the nucleus in mobilized peripheral blood with healthy self-renewal activity (Figure 1(c)) and in peripheral blood from patients with hematopoietic disease that altered their functional self-renewing activity (Figure 1(d)). For these analyses, mobilized peripheral blood and peripheral blood from patients with clinical signs of leukemia were obtained and processed as previously described [18]. Sorted CD34^+^ were deposited in glass by high-speed centrifugation and stained for AHR (clone RPT9) and the nuclear stain DAPI. When possible, a further distinction between long-term (CD34^+^CD38^−^) and short-term (CD34^+^CD38^+^) self-renewing HSC [22] was done using flow cytometry. Intracellular expression of AHR (clone RPT9) was measured as Mean Fluorescent Intensity (MFI) using flow cytometry. Self-renewing capacity of the human hematopoietic cells was measured using xenotransplantation into mice as previously described [23]. Altogether, Figure 1 shows that AHR expression is always present in multiple cell types independent of self-renewal activity. These observations clearly demonstrate that for AHR it is differential signaling and not expression which may drive the regulation of self-renewing activity.

The mammary gland is another tissue where self-renewing stem cells are responsible for maintenance of homeostasis [24]. The evidence supporting AHR involvement in self-renewing activity of breast cells shows that when cultured breast cells were enriched in self-renewing cells, a pathological state was accompanied by changes in AHR responsive genes [25]. The same study showed that SR1 and a related AHR antagonist SR2 increase self-renewal capacity of breast cancer stem cells by upregulating LY6E which is a gene not directly involved in self-renewal but that can alter cell cycling and responses to growth factors. Surprisingly, healthy breast tissue seems to be protected of AHR-mediated carcinogenicity [26] by inhibition of small molecule metabolism-related genes whose action has been proposed to be independent of AHR. Altogether, these results show some interaction of AHR signaling with self-renewing capacity in breast cells but further studies using molecular tools may provide information about the specific players involved.

It has been recently demonstrated that persistent ligand-activation with TCDD in skin stem cells present in the sebaceous glands is responsible for the development of chloracne, a disease characterized by a dysregulation of the terminal differentiation of keratinocytes and metaplasia of sebaceous glands [27]. A complex interaction between transcription factors such as cMyc, Blimp1, and *β*-catenin/TCF (a prominent member of the WNT signaling) stimulate escape from quiescence to a more proliferating status but with a block in terminal sebaceous gland differentiation in favor of proliferation of interfollicular epidermal cells which results in the clinical manifestations of chloracne.

In the mouse, liver cells with multipotent capacity known as oval cells express a functional AHR and its activation has a serious impact on the numbers of these cells [28]. Increased ratios of G0 to G1 in oval cells exposed to the AHR agonist TCDD have been attributed to decreased expression of cyclin D1 and cyclin A and p27 increased expression leading to a block in the phosphorylation of the retinoblastoma protein responsible for the G0 to G1 transition. Whether these observations affect self-renewal capacity directly or just the symmetric divisions, it is not clear but the study provides further support for the flexibility of AHR responses in multiple cell contexts.

A comparison of fetal and adult murine prostate stem cells showed that AHR signaling was enriched together with WNT and HEDGEHOG pathways [29]. This study sorted adult and fetal prostate cells based on expression or lack of Sca-1. As expected, the transcriptional profiles of phenotypically defined stem cells (Sca-1^+^) correlated with in vivo self-renewing capacity of prostate cells. In addition, AHR expression was found to be necessary for the regulation of self-renewal in Sca1^+^ from fetal and adult prostate cells. No further evidence of AHR-HEDGEHOG interaction in other tissues has been described to date. There is some evidence for AHR-NOTCH interactions [30, 31] in the context of the development of the immune system; however, those observations indicate that these interactions affect potency and differentiation capacity rather than self-renewal.

In summary, the disruption of AHR by loss-of-function or ligand binding has repercussions on the self-renewal capacity of stem cells in multiple tissues but it is unclear whether the AHR is a self-renewing pathway to the same extent as the established self-renewal pathways WNT, NOTCH, and HEDGEHOG. Furthermore, the pleiotropic effects of AHR signaling and its ubiquitous tissue expression suggest that AHR's role in self-renewing is being an intermediary of the signaling necessary to support changes in self-renewal.

## 3. The Crosstalk between the AHR and the WNT Self-Renewal Pathway Might Be Directed by Responses to Glucose Availability 

Recent studies using murine embryonic stem cells show that TCDD alters the WNT signaling pathway [32] with devastating effects on cardiomyocyte differentiation. Seminal studies in AHR KO mice, motivated by the cardiac teratogenicity of dioxins, concluded that the embryonic cardiac enlargement observed in AHR KO mice was not associated with an evident cardiac condition but to a subtle dysregulation of insulin that becomes overt with aging [33]. A clear AHR-WNT connection has also been evident when aged HSC from AHR KO mice were studied [34]. These results were consistent with epidemiological data suggesting an increased risk for diabetes in people exposed to potent xenobiotic AHR ligands [35] and a predictive behavior of background levels of xenobiotic AHR ligands other than TCDD for diabetes in the elderly [36]. In addition, loss of HIF1*β*, the nuclear heterodimeric partner of AHR after xenobiotic binding and HIF1*α* in hypoxic environments, mediates human type-2 diabetes [37]. Independent reports suggest that interaction of insulin growth factors (IGF) with their receptors in solid tumors favors self-renewing behavior. Also, TCDD disrupts IGF signaling in breast cancer cells via association of AHR with the CCND1 gene promoter [38]. Interestingly, functional inactivation of IGF and its receptor has been linked to diabetic onset in muscle cells [39]. Altogether, these reports suggested the involvement of AHR signaling in cellular events responsible for glucose homeostasis. A working mechanistic hypothesis that glucose may act as an endogenous ligand of AHR in endothelial cells via binding to responsive elements in the promoter of genes other than the one regulating canonical AHR signaling [40] also shed light on a little known AHR gene target: Thrombospondin-1 (THBS1). It is important to note that when the −5 kb to +2 kb region of the human THBS1 gene promoter was compared to well-known AHR responsive element promoters, there were at least two putative sequences with matching scores close to the training set [41] (Table 1). THBS1 encodes a matrix-related glycoprotein characterized by a TSP1 domain shared by a number of R-spondins [42] that gained interest because of their putative role as adult stem cell growth factors by potentiating WNT signaling [43, 44]. As expected, AHR-dependent dysregulation of WNT signaling via R-spondins has been shown to disrupt tissue regeneration in the zebrafish model [45]. A link between WNT signaling and glucose homeostasis has been extensively addressed in the literature. Broadly speaking, activation of canonical WNT signaling increases lactate production via glucose and glutamine use as carbon sources during proliferating cellular metabolism [46]. In particular, glucose availability within the physiological range can activate WNT signaling when the hexosamine pathway utilizes glucose to produce N-linked glycosylated WNT proteins [47]. Furthermore, WNT dependent stabilization of *β*-catenin in the nucleus of tumor-derived cell lines relies heavily on glucose [48]. Altogether, there is significant evidence that WNT-AHR signaling mediates glucose metabolism [49] that regulates regenerative capacity of stem cells.

**Figure 2:**
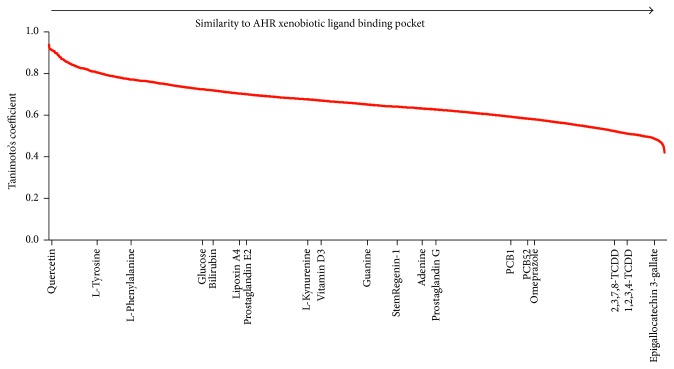
The AHR ligand binding pocket occupied during exposure to toxic xenobiotics such as dioxins (TCDDs) and polychlorinated biphenyls (PCBs) is not likely to accommodate ligands such as SR1, L-kynurenine, or glucose.

Up to date, there are not established endogenous ligands of AHR but there is certain agreement into that different cell types respond differentially to endogenous AHR ligands [50]. A possible rationale for the differences may reside in the use of multiple binding pockets that become available in a cell-specific manner. While there is not enough information to make this type of analysis, a virtual ligand screen was performed to analyze the likelihood of glucose being a ligand for AHR. The web-based software FINDSITEcomb [51] was used to generate a holographic template of a predicted ligand binding pocket for AHR by using information from the CHEMBL database which is a manually curated chemical database of bioactive molecules with drug-like properties maintained by the European Bioinformatics Institute. Since AHR (4M4X) possesses PER-ARNT-SIM ligand binding domains, the software used experimental data for ligands and ligand binding domains from PER-ARNT-SIM-containing templates obtained from Protein Data Bank files that contained the textual files for the three-dimensional structures of EPAS1/ARNT (4GHI), EPAS1/ARNT (3F1N), EPAS1/ARNT (3H7W), Sensory box histidine kinase/response regulator (3MR0), sensor protein (3BWL), and 8-oxoguanine-DNA-glycosylase (3F10). These information allowed obtaining a predicted AHR ligand binding pocket which was then used to perform a virtual ligand screen against 12 271 chemical compounds of the KEGG library including the AHR antagonist SR1. A rank of the compounds based on their similarity to the predicted AHR binding pocket was generated using their Tanimoto's coefficient. Accordingly, the 3D structures of compounds with smaller coefficients are more similar to the binding pocket.  Figure 2 shows that the binding pocket is highly accommodating of xenobiotic compounds; however, glucose together with other proposed endogenous ligands and the antagonist SR1 are unlikely to interact with the same binding pocket. For instance, vitamin D receptor is a good example of a transcription factor with pleiotropic effects that uses more than one ligand binding pocket to initiate signaling [52]. Further studies need to consider whether stem cells may be regulated by AHR ligands via alternative binding pockets which may in turn lead to binding to alternative AHR responsive elements and regulation of different gene promoters.

## 4. AHR Regulates Cellular Energetics

Should glucose act as a ligand of AHR, it would be necessary to identify putative target genes that are regulated by AHR and that can directly intervene in cellular energetics. Spermatocytes in culture exposed to cigarette smoke condensates, known to contain a large number of toxic proinflammatory AHR ligands [53], have a higher glucose uptake but a decreased ATP production [54]. Also, high levels of AHR agonists in human blood serum correlate with a decreased cellular ATP production in myoblasts [55]. Patient-derived AML cells have been shown to have higher oxygen consumption rates [56] which might be consistent with an inefficient oxidative phosphorylation also known as aerobic glycolysis reported in solid tumors where glucose, the main source of energy, is converted to lactate and carbon dioxide due to the hypoxic environment rather than mitochondrial dysfunction [57, 58]. Altogether, the literature suggests that AHR might directly intervene in modulation of cellular energetic usage.

The increased self-renewal by the purine-derivative SR1, an AHR antagonist, measured as numbers of CD34^+^ phenotypically defined HSC, has also been observed in HSC with disrupted self-renewing capacity favoring oxidative phosphorylation of the human bone marrow and peripheral blood-derived cells. While some of the patient samples showed downregulation of CYP1B1, the most consistent observation in these patient samples was the upregulation of the gene COX7B accompanied by changes in cellular metabolism [18]. CYP1B1 is considered the most relevant hematopoietic effector gene of the transcription factor AHR [16] encoding a CYP450 monooxygenase that plays important roles in the metabolism of small molecules involved in cell maintenance and survival, such as steroids [59], but its contribution to changes in cellular energetics is not clear. On the other hand, COX7B is a nuclear encoded gene for one of the subunits of cytochrome c oxidase (COX), the terminal component of the mitochondrial respiratory chain. Interestingly, changes in expression of both COX7B and THBS1 were observed in whole-blood of patients with early onset of acute coronary syndrome [60]. COX7B as well as THBS1 have putative noncanonical AHR responsive elements (Table 1). Interestingly, an alternative AHR responsive element has been described for AHR in breast cancer cells that do not respond to expected AHR ligands but to hypoxic stimuli such as low oxygen tension in culture and cobalt [61]. This study strengthens the argument for the possibility of the extrinsic cues being the most relevant to determine AHR responses since low oxygen tension is a characteristic of stem cells niches. Broadly speaking, self-renewing stem cells depend more on glycolysis than oxidative phosphorylation for ATP production [62] which is considered to be an adaptation to the low oxygen tension of the niches. The metabolic regulation of stem cells has been clearly demonstrated in studies looking at the developmental differences between the highly proliferative fetal liver HSC and adult bone marrow [63]. Similar to what was observed functionally and transcriptionally with SR1 in human HSC irrespective of their intrinsic self-renewing capacity [18], increased self-renewal potential is accompanied by increased expression of genes involved in oxidative phosphorylation for the highly proliferative stem cells. The evidence shown above about an interaction between AHR and WNT signaling in stem cells further supports the involvement of the niches because of the recognized relatively short-range action of WNT proteins in tissue self-renewal [44].

## 5. Future Directions 

Further mechanistic molecular studies are needed to understand the role of AHR regulating self-renewal responses of tissue-resident stem cells niches or whether AHR participates in self-renewal by promoting release of factors by the stem cells themselves. It is expected that the results from those inquiries will place AHR at the center of studies about stem cell metabolism and its impact on aging, regeneration, and cancer stem cells. These studies might have an immediate impact on clinical applications. For instance, they may provide a rationale for the reported clinical improvement of patients with chronic heart failure with low ejection fractions that received bypass grafting plus transplantation of bone marrow-derived stem cells [64, 65] where the presence of stem cells from a different tissue and the absence of its native niche seem to favor regeneration of cardiac tissue.

## Figures and Tables

**Table 1:** Putative AHR responsive elements (AHRE) are present in the −5 kb to +2 kb gene promoter sequence of THBS1 and COX7B. CYP1B1 is shown as a sample gene from the training set, summarized from [41]. ^*∗*^Matching score with respect to a training set of known AHRE.

Gene symbol	mRNA Refseq ID	Number of putative AHRE	Average matching score^*∗*^
THBS1	NM_003246	12	0.7939
COX7B	NM_001866	13	0.7866
CYP1B1	NM_000104	19	0.8203

## References

[1] Mason C., Dunnill P. (2008). A brief definition of regenerative medicine. *Regenerative Medicine*.

[2] Yoo Y. D., Kwon Y. T. (2015). Molecular mechanisms controlling asymmetric and symmetric self-renewal of cancer stem cells. *Journal of Analytical Science and Technology*.

[3] Perry J. M., Li L. (2010). Functional assays for hematopoietic stem cell self-renewal. *Methods in Molecular Biology*.

[4] Ploemacher R. E., Van Der Loo J. C. M., Van Der Sluijs J. P. (1992). In vitro assays for primitive hematopoietic cells. *Blood*.

[5] Taswell C. (1981). Limiting dilution assays for the determination of immunocompetent cell frequencies. I. Data analysis. *The Journal of Immunology*.

[6] Wodarz A. (2005). Molecular control of cell polarity and asymmetric cell division in *Drosophila neuroblasts*. *Current Opinion in Cell Biology*.

[7] Knoblich J. A. (2008). Mechanisms of asymmetric stem cell division. *Cell*.

[8] Wohrer S., Knapp D. J. H. F., Copley M. R. (2014). Distinct stromal cell factor combinations can separately control hematopoietic stem cell survival, proliferation, and self-renewal. *Cell Reports*.

[9] Denison M. S., Vella L. M. (1990). The hepatic Ah receptor for 2,3,7,8-Tetrachlorodibenzo-p-dioxin: species differences in subunit dissociation. *Archives of Biochemistry and Biophysics*.

[10] Denison M. S., Soshilov A. A., He G., DeGroot D. E., Zhao B. (2011). Exactly the same but different: promiscuity and diversity in the molecular mechanisms of action of the aryl hydrocarbon (dioxin) receptor. *Toxicological Sciences*.

[11] Fraccalvieri D., Soshilov A. A., Karchner S. I. (2013). Comparative analysis of homology models of the Ah receptor ligand binding domain: verification of structure-function predictions by site-directed mutagenesis of a nonfunctional receptor. *Biochemistry*.

[12] Dietrich C., Kaina B. (2010). The aryl hydrocarbon receptor (AhR) in the regulation of cell-cell contact and tumor growth. *Carcinogenesis*.

[13] Singh K. P., Wyman A., Casado F. L., Garrett R. W., Gasiewicz T. A. (2009). Treatment of mice with the Ah receptor agonist and human carcinogen dioxin results in altered numbers and function of hematopoietic stem cells. *Carcinogenesis*.

[14] Laiosa M. D., Tate E. R., Ahrenhoerster L. S., Chen Y., Wang D. (2015). Effects of developmental activation of the aryl hydrocarbon receptor by 2,3,7,8-tetrachlorodibenzo-p-dioxin on long-term self-renewal of murine hematopoietic stem cells. *Environmental Health Perspectives*.

[15] van Grevenynghe J., Bernard M., Langouet S., Le Berre C., Fest T., Fardel O. (2005). Human CD34-positive hematopoietic stem cells constitute targets for carcinogenic polycyclic aromatic hydrocarbons. *Journal of Pharmacology and Experimental Therapeutics*.

[16] Boitano A. E., Wang J., Romeo R. (2010). Aryl hydrocarbon receptor antagonists promote the expansion of human hematopoietic stem cells. *Science*.

[17] Smith K. J., Murray I. A., Tanos R. (2011). Identification of a high-affinity ligand that exhibits complete aryl hydrocarbon receptor antagonism. *Journal of Pharmacology and Experimental Therapeutics*.

[18] Casado F. L., Salci K. R., Shapovalova Z., Guezguez B., Collins T. J., Bhatia M. (2016). Expansion of primary human AML by aryl hydrocarbon receptor antagonism minimally affects leukemic transcriptional profiles but alters cellular metabolism. *Journal of Hematology Research*.

[19] Rentas S., Holzapfel N. T., Belew M. S. (2016). Musashi-2 attenuates AHR signalling to expand human haematopoietic stem cells. *Nature*.

[20] Amiel-Pérez J., Casado F. (2015). Stem cells: limitations and opportunities in Peru. *Revista Peruana de Medicina Experimental y Salud Publica*.

[21] Lee J.-H., Mitchell R. R., McNicol J. D. (2015). Single transcription factor conversion of human blood fate to NPCs with CNS and PNS developmental capacity. *Cell Reports*.

[22] Cardoso A. A., Li M.-L., Batard P. (1993). Release from quiescence of CD34+ CD38- human umbilical cord blood cells reveals their potentiality to engraft adults. *Proceedings of the National Academy of Sciences of the United States of America*.

[23] Pearce D. J., Taussig D., Zibara K. (2006). AML engraftment in the NOD/SCID assay reflects the outcome of AML: implications for our understanding of the heterogeneity of AML. *Blood*.

[24] Cicalese A., Bonizzi G., Pasi C. E. (2009). The tumor suppressor p53 regulates polarity of self-renewing divisions in mammary stem cells. *Cell*.

[25] Dubrovska A., Hartung A., Bouchez L. C. (2012). CXCR4 activation maintains a stem cell population in tamoxifen-resistant breast cancer cells through AhR signalling. *British Journal of Cancer*.

[26] Maayah Z. H., Ghebeh H., Alhaider A. A. (2015). Metformin inhibits 7,12-dimethylbenz[a]anthracene-induced breast carcinogenesis and adduct formation in human breast cells by inhibiting the cytochrome P4501A1/aryl hydrocarbon receptor signaling pathway. *Toxicology and Applied Pharmacology*.

[27] Bock K. W. (2016). Toward elucidation of dioxin-mediated chloracne and Ah receptor functions. *Biochemical Pharmacology*.

[28] Faust D., Kletting S., Ueberham E., Dietrich C. (2013). Aryl hydrocarbon receptor-dependent cell cycle arrest in isolated mouse oval cells. *Toxicology Letters*.

[29] Blum R., Gupta R., Burger P. E. (2009). Molecular signatures of prostate stem cells reveal novel signaling pathways and provide insights into prostate cancer. *PLoS ONE*.

[30] Ahrenhoerster L. S., Leuthner T. C., Tate E. R., Lakatos P. A., Laiosa M. D. (2015). Developmental exposure to 2,3,7,8 tetrachlorodibenzo-p-dioxin attenuates later-life Notch1-mediated T cell development and leukemogenesis. *Toxicology and Applied Pharmacology*.

[31] Lee J. S., Cella M., McDonald K. G. (2012). AHR drives the development of gut ILC22 cells and postnatal lymphoid tissues via pathways dependent on and independent of Notch. *Nature Immunology*.

[32] Wang Q., Kurita H., Carreira V. (2016). Ah receptor activation by dioxin disrupts activin, BMP, and WNT signals during the early differentiation of mouse embryonic stem cells and inhibits cardiomyocyte functions. *Toxicological Sciences*.

[33] Thackaberry E. A., Bedrick E. J., Goens M. B. (2003). Insulin regulation in AhR-null mice: embryonic cardiac enlargement, neonatal macrosomia, and altered insulin regulation and response in pregnant and aging AhR-null females. *Toxicological Sciences*.

[34] Singh K. P., Bennett J. A., Casado F. L., Walrath J. L., Welle S. L., Gasiewicz T. A. (2014). Loss of aryl hydrocarbon receptor promotes gene changes associated with premature hematopoietic stem cell exhaustion and development of a myeloproliferative disorder in aging mice. *Stem Cells and Development*.

[35] Tang M., Chen K., Yang F., Liu W. (2014). Exposure to organochlorine pollutants and type 2 diabetes: a systematic review and meta-analysis. *PLoS ONE*.

[36] Lee D.-H., Lind P. M., Jacobs D. R., Salihovic S., van Bavel B., Lind L. (2011). Polychlorinated biphenyls and organochlorine pesticides in plasma predict development of type 2 diabetes in the elderly: the prospective investigation of the vasculature in Uppsala Seniors (PIVUS) study. *Diabetes Care*.

[37] Gunton J. E., Kulkarni R. N., Yim S. (2005). Loss of ARNT/HIF1*β* mediates altered gene expression and pancreatic-islet dysfunction in human type 2 diabetes. *Cell*.

[38] Salisbury T. B., Tomblin J. K., Primerano D. A. (2014). Endogenous aryl hydrocarbon receptor promotes basal and inducible expression of tumor necrosis factor target genes in MCF-7 cancer cells. *Biochemical Pharmacology*.

[39] Fernández A. M., Kim J. K., Yakar S. (2001). Functional inactivation of the IGF-I and insulin receptors in skeletal muscle causes type 2 diabetes. *Genes and Development*.

[40] Dabir P., Marinic T. E., Krukovets I., Stenina O. I. (2008). Aryl Hydrocarbon receptor is activated by glucose and regulates the thrombospondin-1 gene promoter in endothelial cells. *Circulation Research*.

[41] Sun Y. V., Boverhof D. R., Burgoon L. D., Fielden M. R., Zacharewski T. R. (2004). Comparative analysis of dioxin response elements in human, mouse and rat genomic sequences. *Nucleic Acids Research*.

[42] Kamata T., Katsube K.-I., Michikawa M., Yamada M., Takada S., Mizusawa H. (2004). R-spondin, a novel gene with thrombospondin type 1 domain, was expressed in the dorsal neural tube and affected in Wnts mutants. *Biochimica et Biophysica Acta—Gene Structure and Expression*.

[43] Warner M. L., Bell T., Pioszak A. A. (2015). Engineering high-potency R-spondin adult stem cell growth factors. *Molecular Pharmacology*.

[44] Clevers H., Loh K. M., Nusse R. (2014). An integral program for tissue renewal and regeneration: Wnt signaling and stem cell control. *Science*.

[45] Mathew L. K., Simonich M. T., Tanguay R. L. (2009). AHR-dependent misregulation of Wnt signaling disrupts tissue regeneration. *Biochemical Pharmacology*.

[46] Sethi J. K., Vidal-Puig A. (2010). Wnt signalling and the control of cellular metabolism. *Biochemical Journal*.

[47] Anagnostou S. H., Shepherd P. R. (2008). Glucose induces an autocrine activation of the Wnt/*β*-catenin pathway in macrophage cell lines. *Biochemical Journal*.

[48] Chocarro-Calvo A., García-Martínez J. M., Ardila-González S., De la Vieja A., García-Jiménez C. (2013). Glucose-induced *β*-catenin acetylation enhances Wnt signaling in cancer. *Molecular Cell*.

[49] Chafey P., Finzi L., Boisgard R. (2009). Proteomic analysis of *β*-catenin activation in mouse liver by DIGE analysis identifies glucose metabolism as a new target of the Wnt pathway. *Proteomics*.

[50] Opitz C. A., Litzenburger U. M., Sahm F. (2011). An endogenous tumour-promoting ligand of the human aryl hydrocarbon receptor. *Nature*.

[51] Zhou H., Skolnick J. (2013). FINDSITEcomb: a threading/structure-based, proteomic-scale virtual ligand screening approach. *Journal of Chemical Information and Modeling*.

[52] Mizwicki M. T., Keidel D., Bula C. M. (2004). Identification of an alternative ligand-binding pocket in the nuclear vitamin D receptor and its functional importance in 1*α*,25(OH)2-vitamin D3 signaling. *Proceedings of the National Academy of Sciences of the United States of America*.

[53] Baglole C. J., Maggirwar S. B., Gasiewicz T. A., Thatcher T. H., Phipps R. P., Sime P. J. (2008). The aryl hydrocarbon receptor attenuates tobacco smoke-induced cyclooxygenase-2 and prostaglandin production in lung fibroblasts through regulation of the NF-*κ*B family member RelB. *The Journal of Biological Chemistry*.

[54] Omurtag K., Esakky P., Debosch B. J., Schoeller E. L., Chi M. M., Moley K. H. (2015). Modeling the effect of cigarette smoke on hexose utilization in spermatocytes. *Reproductive Sciences*.

[55] Park W.-H., Jun D. W., Kim J. T. (2013). Novel cell-based assay reveals associations of circulating serum AhR-ligands with metabolic syndrome and mitochondrial dysfunction. *Biofactors*.

[56] Škrtić M., Sriskanthadevan S., Jhas B. (2011). Inhibition of mitochondrial translation as a therapeutic strategy for human acute myeloid leukemia. *Cancer Cell*.

[57] Tisdale M. J. (2002). Cachexia in cancer patients. *Nature Reviews Cancer*.

[58] Heiden M. G. V., Cantley L. C., Thompson C. B. (2009). Understanding the Warburg effect: the metabolic requirements of cell proliferation. *Science*.

[59] Jansson I., Stoilov I., Sarfarazi M., Schenkman J. B. (2001). Effect of two mutations of human CYP1B1, G61e and R469W, on stability and endogenous steroid substrate metabolism. *Pharmacogenetics*.

[60] Silbiger V. N., Luchessi A. D., Hirata R. D. C. (2013). Novel genes detected by transcriptional profiling from whole-blood cells in patients with early onset of acute coronary syndrome. *Clinica Chimica Acta*.

[61] Li E.-Y., Huang W.-Y., Chang Y.-C. (2016). Aryl hydrocarbon receptor activates NDRG1 transcription under hypoxia in breast cancer cells. *Scientific Reports*.

[62] Simsek T., Kocabas F., Zheng J. (2010). The distinct metabolic profile of hematopoietic stem cells reflects their location in a hypoxic niche. *Cell Stem Cell*.

[63] Manesia J. K., Xu Z., Broekaert D. (2015). Highly proliferative primitive fetal liver hematopoietic stem cells are fueled by oxidative metabolic pathways. *Stem Cell Research*.

[64] Tian T., Chen B., Xiao Y., Yang K., Zhou X. (2014). Intramyocardial autologous bone marrow cell transplantation for ischemic heart disease: a systematic review and meta-analysis of randomized controlled trials. *Atherosclerosis*.

[65] Rivas-Plata A., Castillo J., Pariona M., Chunga A. (2010). Bypass grafts and cell transplant in heart failure with low ejection fraction. *Asian Cardiovascular and Thoracic Annals*.

